# Langerhans Cell Histiocytosis of the Temporal Bone

**DOI:** 10.31486/toj.19.0032

**Published:** 2020

**Authors:** Scott Mayer, Blake S. Raggio, Adam Master, Nicholas Lygizos

**Affiliations:** ^1^ Department of Surgery, Tulane University Medical Center, New Orleans, LA; ^2^ Department of Otolaryngology, Tulane University Medical Center, New Orleans, LA; ^3^ Department of Otorhinolaryngology, Ochsner Clinic Foundation, New Orleans, LA; ^4^ The University of Queensland Faculty of Medicine, Ochsner Clinical School, New Orleans, LA; ^5^ Ear, Nose and Throat Specialists of Illinois, Chicago, IL; ^6^ Advocate Lutheran General Hospital, Park Ridge, IL

**Keywords:** *Adult*, *histiocytosis–Langerhans cell*, *temporal bone*

## Abstract

**Background:** Langerhans cell histiocytosis (LCH) of the temporal bone is an uncommon disease that primarily affects the pediatric population; fewer than 40 adult cases have been reported in the literature. We present a rare case of LCH of the temporal bone in an adult patient and describe its clinical presentation, histopathologic findings, and management.

**Case Report**: A 21-year-old male presented to the emergency department with progressively worsening right-sided ear pain refractory to outpatient oral antibiotics. Physical examination revealed mastoid tenderness and decreased right-sided hearing. Computed tomography (CT) scan suggested coalescent mastoiditis; the patient responded to inpatient antibiotics and was discharged. He returned 9 days later with persistent symptoms. Repeat CT scan revealed an osteolytic lesion on the temporal bone, and the patient was indicated for surgery. Intraoperative histology was consistent with LCH. Subsequent surveillance magnetic resonance imaging (MRI) suggested persistence of disease, and the patient responded to a course of radiation. Three months following radiotherapy, surveillance MRI and positron emission tomography scans revealed no evidence of recurrent disease.

**Conclusion:** Diagnosis of LCH of the temporal bone is frequently delayed because of misdiagnosis of more common otologic diseases, including otitis media, otitis externa, and mastoiditis. The clinician's index of suspicion for LCH should be high if imaging reveals an osteolytic defect of the temporal bone; confirmation is via immunohistostaining of biopsy samples. The majority of cases respond to surgery, radiation, chemotherapy, or combination therapy, but delays in diagnosis and treatment may increase morbidity. Increased physician awareness of LCH of the temporal bone, particularly among adults, may help to improve patient outcomes.

## INTRODUCTION

Langerhans cell histiocytosis (LCH) is a rare, poorly understood disease characterized by the clonal proliferation of Langerhans cells—lymphoid dendritic cells derived from the bone marrow with antigen-presenting function—outside the dermis.^1^ The incidence of LCH is low, ranging from 1 to 9 per million per year,^2^ with only 25% to 30% of patients experiencing involvement of the temporal bone.^3^

Although all populations can be affected, LCH of the temporal bone primarily affects males^4^ and children (75% to 90% of cases),^2,5^ with a peak incidence at 1 to 3 years of age.^6^ Fewer than 40 adult cases of LCH isolated to the temporal bone have been reported in the literature.^7,8^ We present a rare case of LCH of the temporal bone in an adult patient.

## CASE REPORT

A 21-year-old male presented to the emergency department (ED) with a 3-week history of progressively worsening right-sided ear pain refractory to outpatient oral antibiotics (amoxicillin-sulbactam 875 mg twice daily) prescribed by his primary care physician 4 days earlier. Clinical findings were unremarkable except for exquisite right-sided mastoid tenderness and decreased right-sided hearing. Computed tomography (CT) scan without contrast of the temporal bones revealed opacification of the right mastoid air cells with erosion into the mastoid and sigmoid sinus plate consistent with acute coalescent mastoiditis. Magnetic resonance imaging (MRI) venography revealed no occlusion of the sigmoid sinus. The patient was admitted and started on intravenous (IV) cefepime 1 mg every 6 hours for empiric *Pseudomonas* coverage. The patient's ear pain markedly improved within 24 hours of IV antibiotic administration, and surgical intervention was not planned. He was discharged with oral levofloxacin 750 mg daily for 2 weeks.

The patient missed his 1-week follow-up appointment but presented to a separate quaternary care ED 9 days postdischarge with recurrent right ear pain, mastoid tenderness, and worsened hearing loss. Repeat CT scan identified a lytic lesion of the right mastoid with multiple air-fluid levels of the surrounding mastoid air cells (Figure), inconsistent with the previous diagnosis of coalescent mastoiditis. However, given the patient's lack of systemic symptoms, he was discharged from the ED with a refill of levofloxacin 750 mg and was instructed to urgently follow up with otolaryngology.

**Figure.**
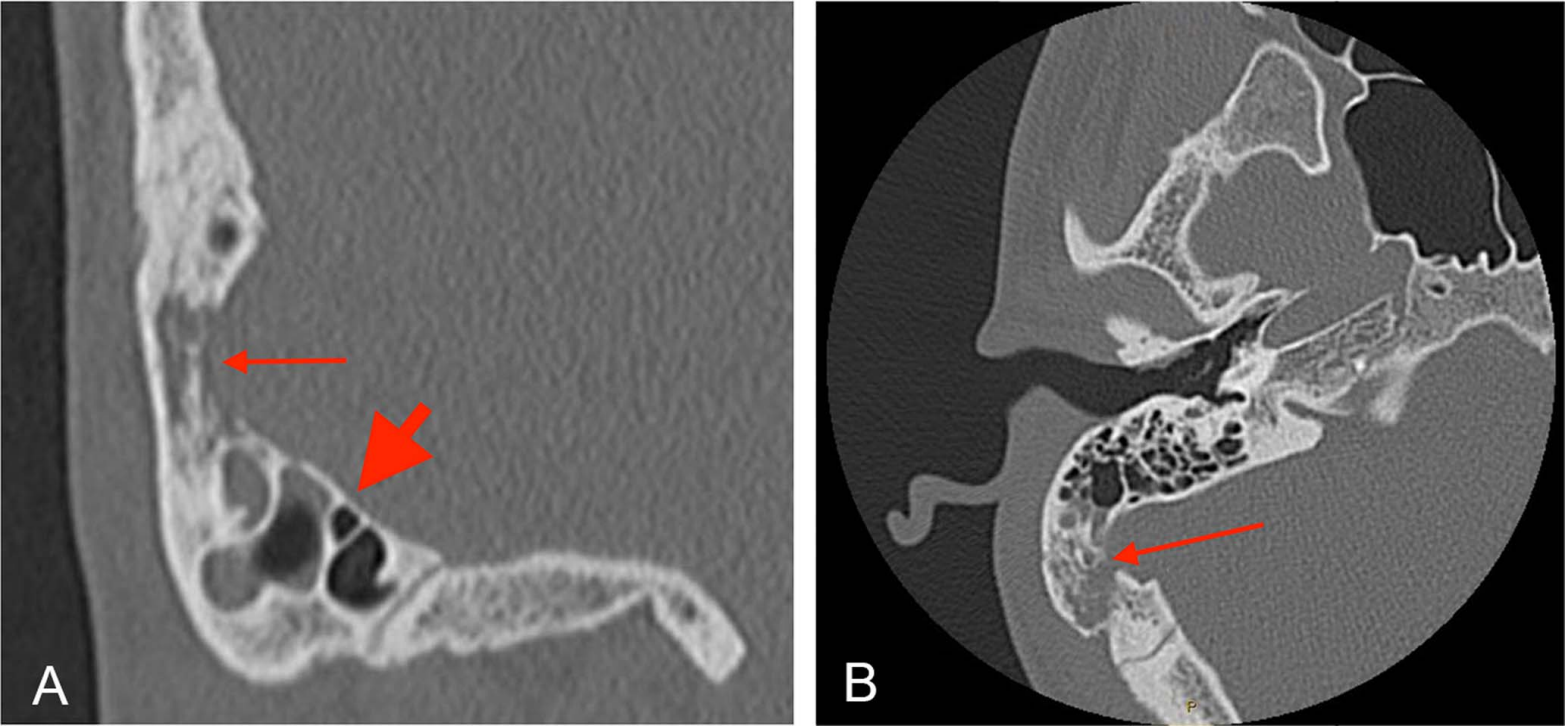
**Initial coronal (A) and axial (B) non–contrast-enhanced computed tomography scans of the right temporal bone show patchy opacification of the posterior right mastoid air cells with erosion of the septa and overlying mastoid cortex. In view A, the thin arrow indicates erosion of bone, and the thick arrow indicates opacification of the mastoid air cells. In view B, the thin arrow indicates erosion of bone.**

Two days later, the patient followed up in otolaryngology clinic. Given his persistent symptoms and the osteolytic lesion seen on the CT scan, the patient consented to mastoidectomy with biopsy. Debridement of the mastoid cavity revealed necrotic bone with extensive granulation tissue throughout the mastoid cortex, as well as a dehiscent but intact sigmoid sinus. Frozen section was consistent with LCH; thus, additional curettage of the remaining granulation tissue was performed. Final diagnosis of LCH was confirmed, with immunohistochemistry showing positivity for CD1a, S100, and CD68. A tympanostomy tube was placed. Intraoperative cultures were negative for growth.

After an uncomplicated 2-day hospital stay, the patient was discharged and instructed to administer ofloxacin 0.3% otic drops (5 drops twice daily) for 1 month. The patient did well until 2 weeks after surgery when he returned to clinic with recurrent pain in the right mastoid. His hearing had returned to normal, but MRI of the brain without contrast and whole-body positron emission tomography (PET)/CT scan suggested residual disease over the sigmoid sinus. The patient was referred to medical and radiation oncology and received a 2-week course of adjunct external beam radiotherapy starting 9 weeks postsurgery at a total dose of 20 Gy, delivered at 2 Gy per fraction.

Three months following radiotherapy and 6 months postoperatively, surveillance MRI and PET scans revealed no evidence of recurrent disease. The patient was instructed to repeat imaging in 6 to 12 months but was lost to follow-up.

## DISCUSSION

LCH is stratified into 2 categories: single-system LCH and multisystem LCH.^9,10^ Single-system LCH is defined by involvement of a single organ system at the time of diagnosis and may feature 1 or multiple lesions.^9^ Unifocal, single-system disease is the most common form of LCH, accounting for 70% of cases.^11^ Multisystem LCH affects 2 or more organ systems. The multisystem category is further stratified into low-risk and high-risk variants according to the organs afflicted. High-risk organs include the liver, lungs, and spleen; involvement of 1 or more of these organs carries the high-risk designation and a greater mortality rate.^9,10^ Former classifications of LCH included eosinophilic granulomatosis, Hand-Schüller-Christian disease, and Letterer-Siwe disease, but these categorizations are outdated and should not be used.^11^ The etiology of LCH is poorly understood, with arguments supporting an autoimmune, neoplastic, or reactive origin of the disease.^12-14^

LCH isolated to the temporal bone is commonly misdiagnosed at initial presentation,^15^ partly because of its rarity but also because its initial clinical presentation is nonspecific—otorrhea, otalgia, postauricular skin rashes, hearing loss, and tissue swelling—and mimics other common otologic pathologies, including otitis media, otitis externa, mastoiditis, ear polyps, and temporal abscess.^16,17^ Laboratory values are also nonspecific, although white blood cell count, erythrocyte sedimentation rate, and C-reactive protein may be elevated.^2,14^ Moreover, an infectious etiology is often suspected because of the transient response to antibiotics, as in our case.^3^ Consequently, diagnosis of isolated LCH of the temporal bone is often delayed, with a median time to diagnosis of 4 months after initial onset of symptoms.^16^

Index of suspicion should arise from unusual findings on imaging. Plain films and CT scans are both considered first-line imaging studies for evaluating LCH of the temporal bone. Plain films are capable of isolating LCH bone lesions that appear as radiolucent, punched-out lesions with well-defined edges.^15^ CT scans are more specific for osteolytic changes and soft tissue densities that manifest around the bony lesion.^3^ MRI is considered second-line imaging and is indicated to evaluate for disease extension—both extracranial and intracranial—or to further characterize soft tissue involvement. PET scans may be used in cases concerning for metastatic lesions.^3^

Ultimately, definitive diagnosis relies on biopsy results, obtained either by fine needle aspirate or excisional biopsy (ie, mastoidectomy). Excisional biopsy is required in 75% of cases.^16^ Grossly, the tumor appears as a friable, polypoid mass in the mastoid cavity with necrosis and bleeding.^5,12,13^ Histologically, LCH consists of multinucleated Langerhans cells with assorted eosinophils, neutrophils, and lymphocytes.^2,5,18^ Definitive diagnosis of LCH, however, requires immunohistochemistry displaying positivity for CD1a and/or langerin (CD207), 2 components of immature dendritic cells.^5,12^ Positivity for S100 can aid in diagnosis but is not specific to LCH.^12^ Electron microscopy reveals characteristic tennis-racquet-shaped organelles known as Birbeck granules, but these may be seen in physiologic Langerhans cells as well. While CD1a and/or langerin are considered pathognomonic for LCH, they also stain positive in Langerhans cell sarcoma, an extremely rare disease with fewer than 100 cases reported.^19^

Treatment of temporal bone LCH is dependent on the stage and severity of disease.^12^ While no consensus treatment regimen is available, several treatment modalities have been proven effective.^17^ Although 10% of isolated temporal bone LCH cases have been reported to resolve spontaneously,^5^ local disease is commonly treated with surgical excision and adjuvant low-dose radiotherapy (10 to 20 Gy) in cases with positive surgical margins.^5^ Multifocal disease should be treated with systemic therapy, including vinblastine (6 mg/m^2^) as the first-line chemotherapy agent with or without concomitant prednisone (40 mg/m^2^/day) therapy for 12 months.^2,18^ Sole radiotherapy (5 to 25 Gy) is also an effective primary treatment option, but an important consideration is that radiation exposure in children significantly increases their risk of developing cancer later in life.^6,17^

Close follow-up with surveillance imaging with MRI and/or PET every 6 months is essential^14^ because up to 50% of patients may experience disease recurrence following initial treatment.^16,13^ Patients treated for multifocal disease have an elevated risk of recurrence (75%) and recur earlier compared to patients with isolated lesions (5 months vs 28 months).^16^ Recurrent disease warrants treatment with multiagent chemotherapy and/or salvage radiation therapy (10 Gy).^16^ In a study by Modest et al, 90% of patients with recurrent LCH survived 42 months after treatment.^16^ Mortality is less frequent in patients with localized (12.5%) vs multifocal (37.5%) temporal bone LCH.^20^

## CONCLUSION

Isolated LCH of the temporal bone is a rare and frequently misdiagnosed disease in adults given its nonspecific clinical presentation. While imaging findings may be helpful, definitive diagnosis relies on tissue biopsy and immunohistochemistry. Treatment of local disease with surgical excision with or without adjuvant radiotherapy and of multifocal disease with chemotherapy has a favorable prognosis; however, close surveillance is critical given the high rate of recurrence. Increased physician awareness of LCH of the temporal bone, particularly in the adult population, may result in timely diagnoses, and consequently, better patient outcomes.

## Notes

This article meets the Accreditation Council for Graduate Medical Education and the American Board of Medical Specialties Maintenance of Certification competencies for Patient Care and Medical Knowledge.
